# SEROLOGICAL CLEARANCE OF HBSAG IN CHRONIC HEPATITIS B: EXPERIENCE IN A UNIVERSITY HOSPITAL

**DOI:** 10.1590/S0004-2803.24612026-020

**Published:** 2026-09-21

**Authors:** Ana Gabriella Gonçalves AMORIM, Alessandra Porto de Macedo COSTA

**Affiliations:** 1 Universidade Federal do Maranhão, Hospital Universitário Presidente Dutra, Departamento de Hepatologia, São Luís, Maranhão, Brasil.

**Keywords:** Chronic hepatitis B, HBsAg clearance, functional cure, Hepatite B crônica, clareamento do HBsAg, cura funcional

## Abstract

**Background:**

Chronic hepatitis B virus (HBV) infection represents a major public health problem due to its high morbidity and mortality associated with liver cirrhosis and hepatocellular carcinoma. Functional cure, characterized by serological clearance of the HBV surface antigen (HBsAg), is the main therapeutic goal, since sterilizing viral eradication is not yet feasible. HBsAg clearance rates vary widely in the literature, and understanding the factors associated with this event is crucial for optimizing patient management.

**Objective:**

To investigate the HBsAg clearance rate and identify the demographic, clinical, and virological factors associated with this event in a cohort of patients with chronic hepatitis B virus infection followed up at a reference center in Northeast Brazil.

**Methods:**

A retrospective cohort study was conducted through a review of the medical records of patients with chronic hepatitis B virus infection treated at a Hepatology Service in Northeast Brazil between January 2015 and October 2025. Clinical, demographic and laboratory variables were analyzed. Statistical analysis used tests such as Mann-Whitney for quantitative variables and Fisher’s exact test for categorical variables, in addition to the construction of Kaplan-Meier curves with the Log-Rank test to evaluate HBsAg clearance. The significance level was set at *P*<0.05.

**Results:**

Of the 600 medical records screened, 299 patients were included in the analysis, 63.2% of whom were women, with a median age of 45 years. During a mean follow-up period of 5.14 years, totaling 1,538 person-years, 12 patients (4%) achieved serological clearance of HBsAg, resulting in an annual rate of 0.78%. Statistical analysis revealed significant associations between HBsAg clearance and advanced age (*P*=0.008) and lower HBV-DNA levels (*P*=0.0156).

**Conclusion:**

The annual rate of HBsAg serological clearance in this cohort was 0.78%. Advanced age and low viral load levels were significantly associated with functional cure. These findings enrich the knowledge about the natural history of chronic hepatitis B in the region and reaffirm the importance of viral suppression in disease control.

## HIGHLIGHTS

• The factors that determine HBsAg seroclearance in patients with chronic hepatitis B are not yet fully established.

• A retrospective cohort study of 299 patients with chronic hepatitis B sought to identify factors associated with functional cure.

• The annual HBsAg clearance rate was 0.78% and was correlated with advanced age and low or undetectable HBV-DNA levels.

• Periodic HBsAg determination and monitoring of HBV-DNA levels are strategies for detectingfunctional cure.

## RESUMO

**Contexto:**

A infecção crônica pelo vírus da hepatite B (HBV) representa um grave problema de saúde pública devido à sua elevada morbidade e mortalidade associadas à cirrose hepática e ao carcinoma hepatocelular. A cura funcional, caracterizada pela depuração sorológica do antígeno de superfície do HBV (HBsAg), é o principal objetivo terapêutico, visto que a erradicação viral esterilizante ainda não é viável. As taxas de clareamento do HBsAg são amplamente variáveis na literatura, e a compreensão dos fatores associados a esse evento é crucial para otimizar o manejo dos pacientes.

**Objetivo:**

Investigar a taxa de clareamento do HBsAg e identificar os fatores demográficos, clínicos e virológicos associados a esse evento em uma coorte de pacientes com infecção crônica pelo vírus da hepatite B, acompanhados em um centro de referência no Nordeste do Brasil.

**Métodos:**

Foi conduzido um estudo de coorte retrospectivo, por meio da revisão de prontuários de pacientes com infecção crônica pelo vírus da hepatite B atendidos em um Serviço de Hepatologia no Nordeste do Brasil, entre janeiro de 2015 a outubro de 2025. Foram analisadas variáveis clínicas, demográficas e laboratoriais. A análise estatística empregou testes como Mann-Whitney para variáveis quantitativas e exato de Fisher para variáveis categóricas, além da construção de curvas de Kaplan-Meier com teste Log-Rank para avaliar o tempo até o clareamento do HBsAg. O nível de significância adotado foi de *P*<0,05.

**Resultados:**

Dos 600 prontuários triados, 299 pacientes foram incluídos na análise, sendo 63,2% mulheres e mediana da idade de 45 anos. Durante um período de acompanhamento médio de 5,14 anos, totalizando 1538 pessoas-ano, 12 pacientes (4%) alcançaram o clareamento sorológico do HBsAg, resultando em uma taxa anual de 0,78%. A análise estatística revelou associações significativas entre o clareamento do HBsAg e a idade avançada (*P*=0,008) e níveis mais baixos de HBV-DNA (*P*=0,0156).

**Conclusão:**

A taxa anual de clareamento sorológico do HBsAg nesta coorte foi de 0,78%. Idade avançada e níveis baixos de carga viral foram significativamente associados à cura funcional. Esses achados enriquecem o conhecimento sobre a história natural da hepatite B crônica na região e reafirmam a importância da supressão viral para o controle da doença.

## INTRODUCTION

Chronic hepatitis B virus (HBV) infection represents a significant public health problem due to its high morbidity and mortality, affecting approximately 254 million people worldwide, with an estimated 1.1 million deaths caused by the virus, associated with liver cirrhosis and hepatocellular carcinoma (HCC), which can take decades to manifest^1^. In Brazil, it is estimated that 1.1 million people have chronic HBV infection. In 2021, the rate was 3.4 cases per 100,000 inhabitants. Fortunately, rates have decreased in people under 40 years of age, due to the introduction of the vaccination schedule from 1990 onward^2^.

HBV is transmitted through contact with infected blood and bodily fluids. The main routes of transmission are unprotected sexual intercourse with an infected person; from an infected mother to her baby during pregnancy or childbirth; sharing syringes and needles; sharing personal hygiene items; tattooing, piercing, and dental, surgical, medical and hemodialysis procedures; close person-to-person contact (cuts, wounds, and skin breaks); and blood transfusions (more related to the period before 1993)^1^.

HBV is an enveloped, partially double-stranded DNA hepatotropic virus belonging to the Hepadnaviridae family. To date, ten genotypes (A to J) have been described, with genotypes A, D and F predominating in Brazil^3^. Some genotypes have more frequent clinical associations with liver cirrhosis and HCC, such as genotypes C and D^4^ ^,^ ^5^.

Acute HBV infection has a mean incubation period of 75 days, predominantly manifesting as a subclinical and self-limiting condition in up to 70% of patients. Although jaundice occurs in about 30% of cases, progression to severe liver failure is rare (less than 1%). Functional cure is observed in 95% of adults. However, the persistence of HBsAg for more than six months characterizes chronic hepatitis B, the risk of which is inversely proportional to age, reaching 90% in newborns and falling to less than 10% in healthy adults. In the chronic stage, progression to cirrhosis occurs in up to 25% of cases, significantly increasing the annual risk of liver decompensation and hepatocellular carcinoma, especially in already cirrhotic patients^6^.

The natural history of chronic HBV infection reflects the dynamic interaction between viral replication and host immune response, and virological markers such as hepatitis B virus E antigen (HBeAg) status, serum HBV DNA, and alanine aminotransferase (ALT) levels are used to determine the stage of chronic infection and predict prognosis^7^ ^,^ ^8^.

Sterilizing cure of chronic hepatitis B is not possible due to the presence of integrated HBV DNA and the long half-life of covalently closed circular DNA (cccDNA). Therefore, the therapeutic goal for new treatments of chronic hepatitis B is to achieve functional cure, i.e., HBsAg seroconversion^9^ ^-^ ^11^. Functional cure, characterized by serological clearance of HBsAg, means sustained immunological control of HBV and is associated with better clinical outcomes, with a lower risk of decompensated cirrhosis, HCC, and liver-related death^12^.

HBsAg seroconversion can occur spontaneously, but reported rates have been variable. Previous cohort studies have shown that the annual rate of HBsAg seroconversion can range from a minimum of 1.02% to a maximum of 2.38%^12^ ^-^ ^14^ and is affected by several patient characteristics such as sex, age, cirrhosis, HBeAg status, HBV DNA level and serum HBsAg level^15^. Furthermore, reported rates of HBsAg seroconversion with currently available treatments are low^16^ ^,^ ^17^.

Functional cure of hepatitis B is a multifactorial event, influenced by viral, clinical, and immunological host variables. Although current therapies have modest success rates, advances in viral biology and immunopathogenesis have driven innovative strategies to broaden this ideal outcome. Understanding the immunological mechanisms of HBsAg loss is fundamental to the development of curative therapies, aligning with the WHO goal of eliminating viral hepatitis as a public health problem by 2030^1^.

However, the rarity of HBsAg seroconversion makes it difficult to accurately determine its incidence and predictors. Given this gap, this study aims to investigate the HBsAg clearance rate and identify the factors associated with this event in patients followed at a reference center in Northeast Brazil.

## METHODS

This is a retrospective, cohort study conducted through the review of medical records of patients with chronic HBV infection followed up at the Hepatology Service of University Hospital of Federal University of Maranhão, in São Luís, capital of the state of Maranhão, Brazil, from January 2015 to October 2025. Inclusion criteria were patients of both sexes; minimum age of 14 years and presence of reactive HBsAg for more than 6 months. Excluded from the study were patients post-liver transplant; those who arrived at the service already diagnosed with functional cure; individuals with fewer than two HBV-DNA determinations; patients who had undetectable HBV-DNA but without HBsAg to confirm functional cure; and cases with incomplete medical records, i.e., without the necessary information for proper analysis.

The study was conducted in accordance with Resolution No. 466 of the National Health Council, dated 12/12/2012, which regulates research involving human beings in Brazil, and was approved by the Research Ethics Committee of Hospital Presidente Dutra (CAAE: 91572425.2.0000.5086). Given that the research was based on a review of medical records, a waiver of the application of the Informed Consent Form was requested. 

### Data collection

Data collection was performed through a systematic review of the medical records of eligible patients during the study period, and clinical, demographic, and laboratory variables were analyzed. Sociodemographic data included sex and age at the first consultation, and age was categorized as 39 or younger, 40 to 49, 50 to 59, and 60 or older. Comorbidities considered included type 2 diabetes mellitus (DM2), systemic arterial hypertension (SAH), obesity, hepatic steatosis on ultrasound, any type of alcohol consumption and co-infections with other viruses (HCV, HDV and HIV). The biochemical and virological laboratory tests evaluated were serum levels of ALT, HBeAg and HBV-DNA levels. The presence of HCC and the staging of liver disease were also evaluated by transient elastography (FibroScan®) and/or biopsy, performed within one year of the first medical appointment, considering the stages of fibrosis (F0 to F3) and the presence of cirrhosis (F4).

Patients with detectable HBV viral load above 10 IU/mL were considered reactive HBsAg. For ALT, values were considered normal if equal to or less than 25 U/L for women and 35 U/L for men^2^. For patients who received treatment, the antiviral prescribed, date of treatment initiation and duration of treatment until HBsAg clearance and the virological response were analyzed. The diagnosis of liver cirrhosis for patients who had not undergone transient elastography or liver biopsy was made according to clinical, laboratory, and imaging criteria (ultrasound, computed tomography, abdominal magnetic resonance imaging, or upper digestive endoscopy). Obesity was defined according to WHO criteria^18^, which define obesity as a body mass index (BMI) ≥30 kg/m^2^.

### Statistical analysis

All collected data were organized in a Microsoft Excel® spreadsheet. Categorical variables were reported as percentages and numerical variables were presented as median and interquartile range (IQR), since non-parametric methods were used after applying the Shapiro-Wilk test. The Mann-Whitney test was conducted to assess associations between HBsAg clearance and quantitative variables, such as age and HBV-DNA levels. Fisher’s exact test was used to identify associations between HBsAg clearance and categorical variables.

Taking into account models observed in the literature, this study calculated the person-year value as the sum of the years elapsed from the first medical appointment until the clearance of HBsAg or the last follow-up visit, for each patient, whichever occurred first. The annual rates of serological clearance were calculated by dividing the number of cases by the person-year value.

Kaplan-Meier curves were constructed to analyze the relationship between variables and the time elapsed until HBsAg clearance. The comparison of the time distribution until the event between different groups was performed using the Log-Rank test, in order to identify statistically significant differences between groups.

The significance level was set at 5%, and a *P*-value <0.05 was considered statistically significant. The analyses were performed using RStudio v. 2025.09.2+418 software and functions contained in the Survivor and Survminer packages.

## RESULTS

**FIGURE 1:**
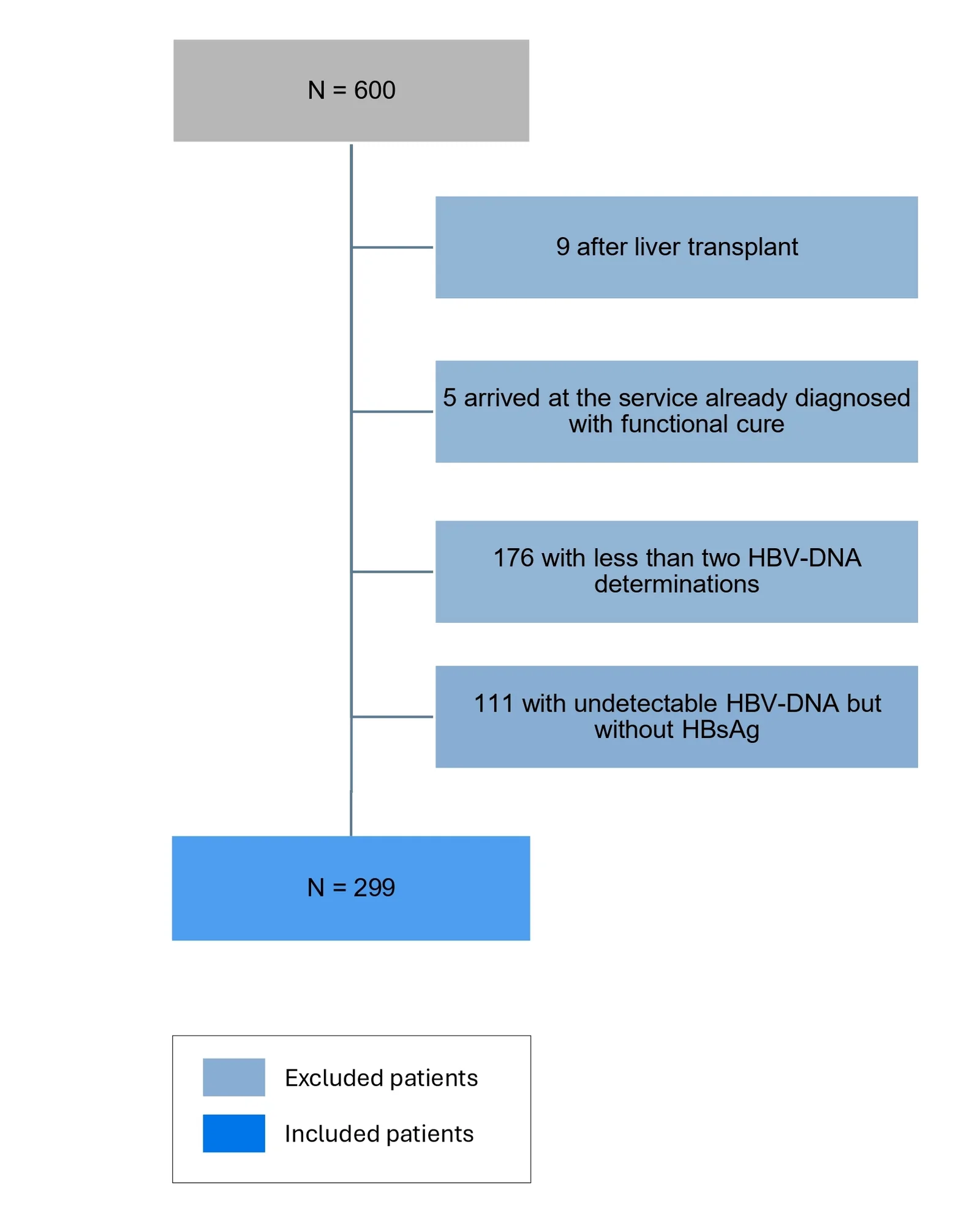
Flowchart showing the inclusion of patients with chronic hepatitis B in the study (in absolute numbers).

During the study period, 600 patients with chronic hepatitis B were attended to at the Hepatology Service of the Presidente Dutra Hospital. Based on the established exclusion criteria, 299 patients were included in the study (Figure 1).

HBV-DNA: hepatitis B viral load. HBsAg: hepatitis B virus surface antigen.

Of the 299 patients, 189 were female (63.2%) and the median age of the patients was 45 years (15-85). Regarding alcohol consumption history, 128 patients (42.8%) had a record of consumption in their medical records, regardless of the amount. One hundred and forty-six patients (48.8%) were non-drinkers and 25 patients (8.4%) had no record of alcohol use. Among associated comorbidities, 72 patients (24%) had systemic arterial hypertension, 41 (13%) had a record of type 2 diabetes, and 51 (17%) had some degree of obesity. Ten patients had a record of both diabetes and obesity. As for the patients who underwent ultrasound, hepatic steatosis was detected in 85 cases (28.4%). Regarding co-infections, 241 patients were tested for anti-HCV, with no positive cases. Anti-HIV testing was performed on 182 patients, with a positive result in only one case.

Concerning the assessment of the degree of hepatic fibrosis, approximately 50% of the sample (151 patients) underwent liver elastography using FibroScan® within the first year of follow-up, with 136 (90%) presenting with stage F0/F1 fibrosis, seven (5%) with F2, four (2.5%) with F3, and four (2.5%) with stage F4. Fifteen patients underwent liver biopsy (nine with stage F0, five with F1, and only one with F2). According to clinical, laboratory, and/or imaging evaluation, the majority of patients (86.9%) did not present any evidence of cirrhosis. Thirty-nine patients (13%) were already cirrhotic at the beginning of follow-up or progressed to it during the follow-up period. Eight patients with cirrhosis (20%) progressed to HCC.

A total of 262 patients (87.6%) had non-reactive HBeAg, while only 28 (9.3%) had reactive HBeAg. HBeAg was not determined in nine patients. Regarding initial HBV-DNA levels, 201 patients (67.2%) had less than 2.000 IU/mL, 56 patients (18.7%) had between 2.000 and 20.000 IU/mL, and 40 patients (13.4%) had above 20.000 IU/mL. Concerning clinical phases, the majority of patients (69.2%) were in the chronic infection phase with non-reactive HBeAg, previously referred to as the inactive carrier phase. The clinical phase of infection could not be determined in 62 patients. Overall, 213 patients (71.2%) received no treatment, while 86 patients (28.7%) were treated with an antiviral. Of those who underwent treatment, 51 patients (59.3%) had undetectable HBV-DNA during follow-up. The demographic, virological, and clinical characteristics of the 299 patients with chronic hepatitis B virus infection are shown in Table 1.

**TABLE 1:** Demographic, Virological And Clinical Characteristics Of The 299 Patients With Chronic Hepatitis B.

Characteristics	N=299	
**Sex**, no. (%)		
Female	189	(63,2)
Male	110	(36,8)
**Age**, median (min-max)	45	(15-85)
**Age**, no. (%)		
≤39 years old	121	(40,5)
40 to 49 years old	70	(23,4)
50 to 59 years old	60	(20,1)
≥60 years old	48	(16,1)
**Comorbidities**, no. (%)		
Type 2 Diabetes Mellitus	41	(13,7)
Systemic arterial hypertension	72	(24,1)
Obesity	51	(17,1)
Hepatic steatosis	85	(28,4)
**Co-infections**, no. (%)		
HIV	1	(0,3)
HCV	0	(0,0)
**Cirrhosis**, no. (%)	39	(13,0)
**HCC**, no. (%)	8	(2,7)
**Reactive HBeAg,** no. (%)	28	(9,4)
**HBV-DNA (UI/mL),** median (min-max)	729	(10-578.10^6^)
HBV-DNA*, no. (%)		
Undetectable	58	(19,4)
<2.000 UI/mL	201	(67,2)
2.000 to 20.000 UI/mL	56	(18,7)
>20.000 UI/mL	40	(13,4)
Missing data	2	(0,7)
Alanine aminotransferase*, no. (%)		
>25 UI/L (women)	53	(17,7)
>35 UI/L (men)	49	(16,4)
Clinical phase		
Reactive HBeAg chronic infection	9	(3,0)
Reactive HBeAg chronic hepatitis	10	(3,3)
Non-reactive HBeAg chronic infection	209	(69,9)
Non-reactive HBeAg chronic hepatitis	9	(3,0)
Missing data	62	(20,7)
**Antiviral treatment**, no. (%)	86	(28,8)

*HBV-DNA and alanine aminotransferase values refer to the first available measurements.

HIV: human immunodeficiency virus. HCV: hepatitis C virus. HCC: hepatocellular carcinoma. HBeAg: hepatitis B virus E antigen. HBV-DNA: hepatitis B viral load.

In terms of HBsAg clearance, of the 299 patients, 12 (4%) became negative for the serological marker. A total of 1538 person-years were calculated, with a mean follow-up of 5.14 years, and 12 cases of HBsAg loss, resulting in an annual serological clearance rate of 0.78%.

A statistically significant association was observed between age and HBsAg clearance, with higher proportions of functional cure in older patients. The median age of individuals who achieved functional cure was 52.5 years. When categorizing by age, the 60 years and older age group showed the highest rate of functional cure, representing 41.7% of the total events. In contrast, the group under 39 years of age, although the largest in the total sample, accounted for only 16.7% of clearance cases. Additionally, lower viral load levels were associated with HBsAg loss. The group that achieved functional cure had a considerably lower median viral load (129.5 IU/mL) compared to the group without cure (803 IU/mL). Characteristics such as sex, presence of liver cirrhosis, and antiviral treatment did not show statistically significant correlations with HBsAg negativity. The factors associated with HBsAg clearance in the 299 patients with chronic hepatitis B are shown in Table 2.

**TABLE 2:** Factors Associated With HBsAg Clearance In 299 Patients With Chronic Hepatitis B.

Characteristics	HBsAg clearance				***P*-value**
	Yes (N=12)		No (N=287)		
**Sex**, no. (%)					
Female	6	(50,0)	183	(63,8)	0,3683
Male	6	(50,0)	104	(36,2)	
**Age**, median (IQR)	52,5	(18,5)	43	(21)	0,0139
**Age**, no. (%)					0,0080
≤39 years old	2	(16,7)	119	(41,5)	
40 to 49 years old	1	(8,3)	69	(24,0)	
50 to 59 years old	4	(33,3)	56	(19,5)	
≥60 years old	5	(41,7)	43	(15,0)	
**Comorbidities**, no. (%)					
Type 2 Diabetes Mellitus	3	(25,0)	38	(13,2)	0,2180
Systemic arterial hypertension	6	(50,0)	66	(23,0)	0,0426
Obesity	2	(16,7)	49	(17,1)	1,0000
Hepatic steatosis	5	(41,7)	80	(27,9)	0,3317
**Co-infections**, no. (%)					
HIV	0	(0,0)	1	(0,3)	1,0000
HCV	0	(0,0)	0	(0,0)	Indet.
**Cirrhosis**, no. (%)	1	(8,3)	38	(13,2)	1,0000
HCC, no. (%)	0	(0,0)	8	(2,8)	1,0000
**Reactive HBeAg,** no. (%)	0	(0,0)	28	(9,8)	0,6121
**HBV-DNA (UI/mL),** median (IQR)	129,5	(498)	803	(3930)	0,0156
**Undetectable HBV-DNA**, no. (%)	12	(100)	46	(16,0)	0,0000
HBV-DNA*, no. (%)					0,2598
<2.000 UI/mL	10	(83,3)	191	(66,6)	
2.000 to 20.000 UI/mL	1	(8,3)	55	(19,2)	
>20.000 UI/mL	1	(8,3)	39	(13,6)	
Missing data	0	(0,0)	2	(0,7)	n/a
Alanine aminotransferase*, no. (%)					
>25 UI/L (women)	2	(16,7)	51	(17,8)	0,6735
>35 UI/L (men)	1	(8,3)	48	(16,7)	0,2229
**Clinical phase**					0,6582
Reactive HBeAg chronic infection	0	(0,0)	9	(3,1)	
Reactive HBeAg chronic hepatitis	0	(0,0)	10	(3,5)	
Non-reactive HBeAg chronic infection	11	(91,7)	198	(69,0)	
Non-reactive HBeAg chronic hepatitis	1	(8,3)	8	(2,8)	
Missing data	0	(0,0)	62	(21,6)	n/a
**Antiviral treatment**, no. (%)	6	(50)	80	(27,9)	0,1103

*HBV-DNA and alanine aminotransferase values refer to the first available measurements.

HBsAg: hepatitis B virus surface antigen. HIV: human immunodeficiency virus. HCV: hepatitis C virus. HCC: hepatocellular carcinoma. HBeAg: hepatitis B virus E antigen. HBV-DNA: hepatitis B viral load.

**FIGURE 2:**
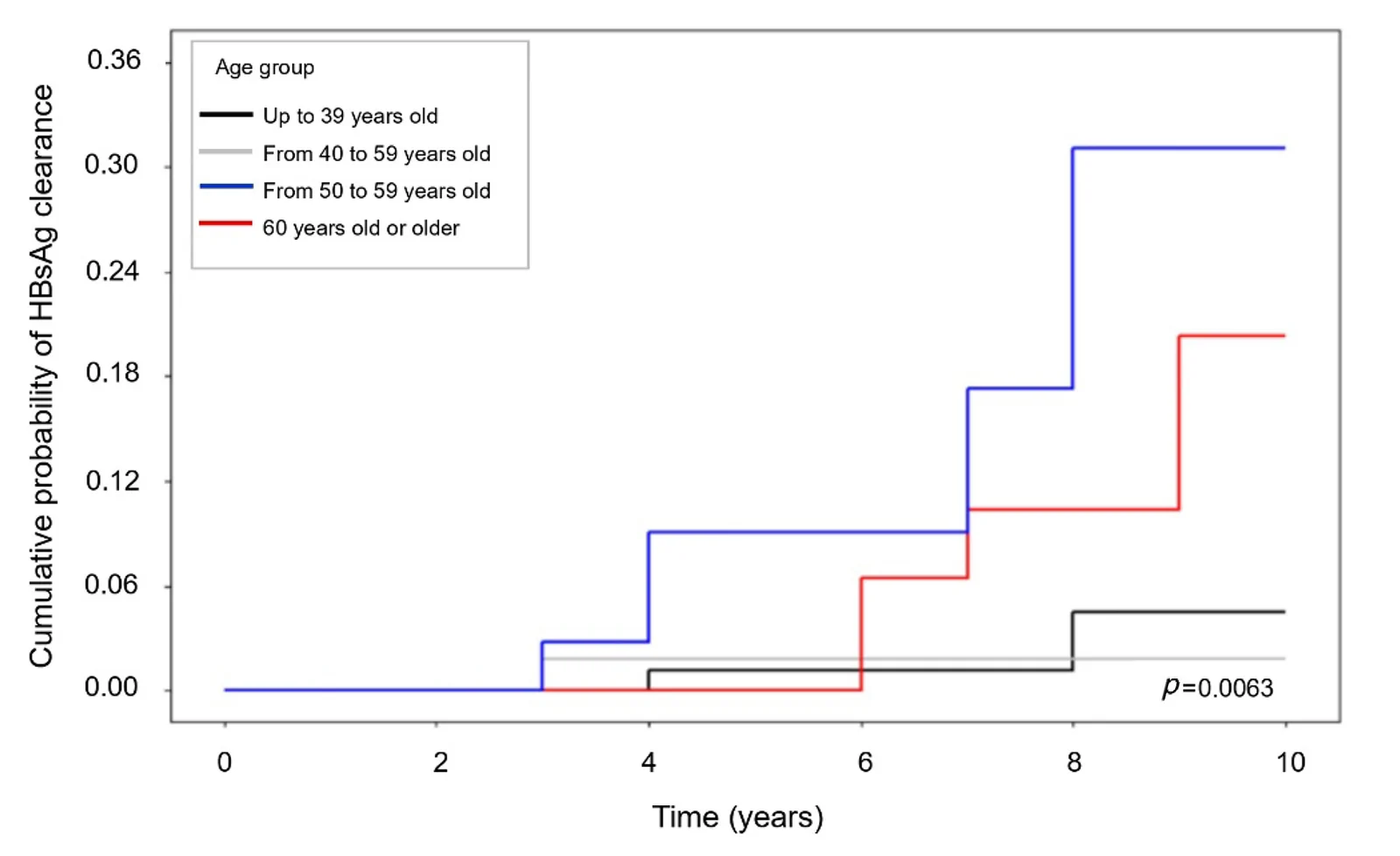
Cumulative incidence of HBsAg clearance according to age group.

**FIGURE 3:**
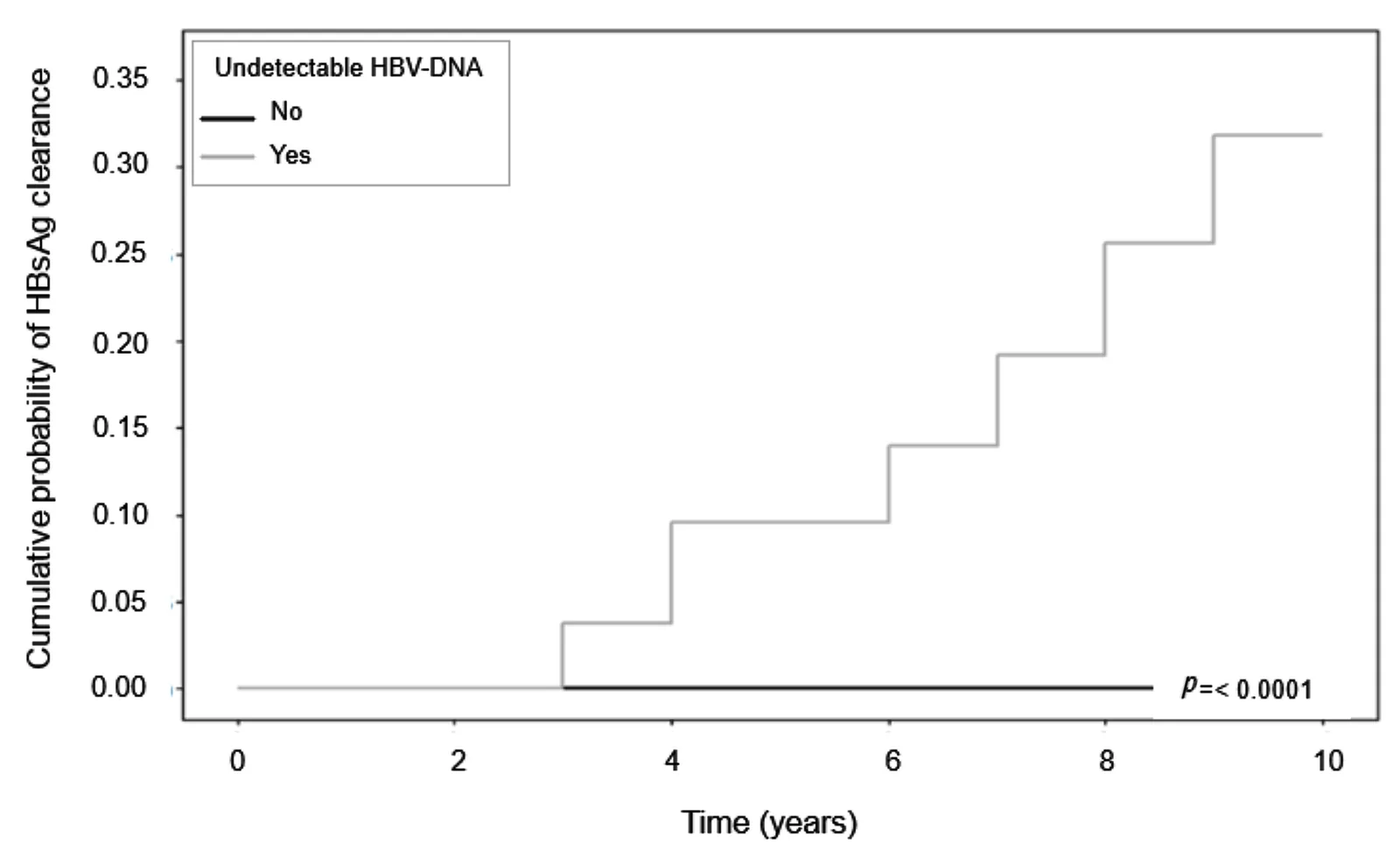
Cumulative incidence of HBsAg clearance according to undetectable HBV-DNA.

The variables associated with HBsAg clearance were plotted on cumulative incidence curve graphs. Patients of older age and with undetectable HBV-DNA achieved functional cure more quickly, as shown in Figures 2 and 3, respectively. Due to the limited number of events (n=12), multivariate analysis by logistic regression was not performed to avoid the risk of overfitting the statistical model.

HBsAg: hepatitis B virus surface antigen.

HBsAg: hepatitis B virus surface antigen. HBV-DNA: hepatitis B viral load.

## DISCUSSION

Serological clearance of HBsAg represents functional cure of chronic HBV infection, a clinical outcome of great importance, associated with a lower risk of progression to cirrhosis, HCC and liver disease-related mortality. This study aimed to investigate the rate of HBsAg clearance and the factors associated with this event in a cohort of patients followed at a reference center in Northeast Brazil. The findings provide valuable information on the natural history of chronic HBV infection in a regional setting, contributing to the understanding of the mechanisms underlying functional cure.

In this cohort, a predominance of female patients (63.2%) was observed, differing from the literature, which reports a higher prevalence of chronic HBV infection in males: only 41.3% and 39.4% were women in a Brazilian cohort^3^ and in a worldwide cohort^15^, respectively. This finding may reflect regional patterns of access to health services or the search for care at a referral center. It is important to note, however, that in the context of this study, sex was not statistically associated with HBsAg clearance (*P*=0.3683), suggesting that, for this specific outcome, gender disparity in the sample was not a determining factor.

The median age found was 45 years, similar to the median found in the meta-analysis published in 2019 by Yeo et al.^12^, although slightly higher than in a Brazilian multicenter study from 2017^3^, with a median of 43.4 years and a multinational cohort^15^ published more recently, in 2023, with a median of 38 years.

Most patients (69.2%) were in the non-reactive HBeAg chronic infection phase at the start of follow-up, characterized essentially by low HBV-DNA levels (<2.000 IU/mL), normal ALT and absence of signs suggestive of liver inflammation and/or fibrosis^10^ ^,^ ^19^. This is a common profile in populations of chronic HBV carriers around the world^7^ ^,^ ^20^.

Regarding the presence of liver cirrhosis, only 13% of patients were cirrhotic or progressed to it during the follow-up period, a higher proportion than that seen in a multinational cohort^21^ published in 2021, with 6.5% of cirrhosis cases in patients followed for just over 8 years. However, the prevalence of cirrhosis in cohorts of patients with chronic HBV infection may vary depending on the inclusion criteria and follow-up time. The presence of cirrhosis is a critical factor for the management of hepatitis B and for the risk stratification of HCC, which was observed in 2.7% of the total sample and in 20% of cirrhotic patients who progressed to HCC.

Concerning the incidence of functional cure during follow-up, 12 cases (4%) were found, with an annual rate of 0.78% - a lower rate than those reported in multicenter studies, which range from 1.02% to 2.38%^12^ ^-^ ^14^. The rarity of this event underscores the complexity of chronic HBV infection. Sterilizing cure remains unattainable due to the persistence of integrated HBV DNA and cccDNA, making functional cure the most clinically relevant therapeutic goal for new treatments^9^. The observation that the annual clearance rate in this cohort is lower than that found in the literature reinforces the relevance of further investigating specific factors that may influence this progression in the population of the region.

The statistically significant association between age group and HBsAg clearance is a finding already demonstrated in the literature^12^ ^,^ ^14^ ^,^ ^24^. Patients who achieved functional cure had a higher median age (52.5 years) compared to the group without cure (43 years). More specifically, the age group of 60 years or older represented 41.7% of the total functional cure events (*P*=0.008), despite being a smaller group in the total sample. In contrast, the group under 39 years of age, the most populous in the study, contributed only 16.7% of the clearance cases. This finding indicates that this event tends to be late and reflects a prolonged dynamic between the immune system and the virus, favored by the maintenance of low levels of viral replication over the years.

The strong association between lower HBV viral load levels and HBsAg clearance is one of the cornerstones for understanding the natural history of hepatitis B. In the present study, more than half of the patients (59.3%) who were undergoing treatment progressed to undetectable HBV-DNA. It is known that low or undetectable HBV-DNA levels correlate with a greater chance of functional cure^12^ ^,^ ^14^ ^,^ ^24^, as confirmed by this cohort. The median HBV-DNA in patients who progressed to HBsAg clearance was considerably lower compared to the group without functional cure (129.5 IU/mL vs 803 IU/mL; *P*=0.0156). Notably, 100% of patients with functional cure presented undetectable viral load during follow-up (*P*=0.0000), a striking contrast to the 16% rate of undetectable HBV-DNA in the group without cure. This result reinforces the premise that effective viral suppression, whether spontaneous or treatment-induced, is a fundamental precondition for HBsAg loss^8^ ^,^ ^24^. Low viral replication allows for a greater likelihood of sustained immune control, leading to antigen clearance. This finding is largely consistent with the literature, which frequently identifies viral suppression as one of the most robust predictors of HBsAg seroclearance^12^ ^,^ ^14^ ^,^ ^24^.

Through the analysis of the cumulative probability of HBsAg clearance over a 10-year follow-up, it was observed that older patients with undetectable HBV-DNA achieved functional cure more quickly (*P*=0.0063 and *P*<0.0001, respectively) compared to younger patients with detectable viral load levels.

Based on a detailed analysis of functional cure cases, half of the patients were undergoing antiviral treatment. Therefore, it was not possible to correlate the loss of HBsAg with medication use with statistical significance. The results indicate that, in the context of this study, functional cure was predominantly an event in the natural history of the disease, influenced by host factors and viral load, regardless of the use of antiviral therapy.

Current antiviral treatments, while highly effective in viral suppression, rarely directly induce HBsAg loss. The main function of the drugs is to control viral replication and prevent disease progression, not necessarily to eradicate the virus and achieve HBsAg clearance in most patients^16^. The literature shows that functional cure rates in patients using antiviral therapies are not very high, with overall rates rarely exceeding 10% in medium-term follow-up^16^. A multinational randomized trial, published in 2018, concluded that clearance rates were higher in patients on combined antiviral therapy [nucleos(t)ide analog tenofovir disoproxil (TDF) with pegylated interferon alpha (PegIFα)], compared to monotherapy with PegIFα^17^. However, other studies report higher rates for patients on PegIFα monotherapy when compared with the use of nucleos(t)ides such as TDF or entecavir (ETV)^22^ ^,^ ^23^. Regarding the use of ETV or TDF, the literature is controversial. In the Jeng et al. cohort^13^, also published in 2018, clearance was higher in patients treated with ETV; in the RETRACT-B study, published in 2022^24^, the findings were opposite, with higher rates with TDF treatment. Recent studies have observed higher rates of functional cure after discontinuation of antiviral treatment, particularly in non-cirrhotic patients^24^ ^-^ ^26^. In the present cohort, since there was no statistical significance regarding the use of antiviral therapy, the drug classes used by the patients was not explored in detail.

Regarding comorbidities, only systemic arterial hypertension (SAH) presented a *p*-value of 0.0426, close to the significance threshold, suggesting a possible direction for future investigations, although it was not considered statistically significant in this study. A possible explanation is due to the fact that older patients have a higher risk of being hypertensive when compared to younger patients, with the correlation affecting on age and not on the comorbidity itself.

Although all patients who achieved functional cure were non-reactive HBeAg, there was no statistical significance due to the low proportion of patients with this reactive biomarker in the population of chronic HBV infection carriers (9.4%). Another correlation that did not show statistical significance was in relation to liver cirrhosis. Of the patients who showed HBsAg clearance, only one was cirrhotic. The sample of cirrhotic patients was low in both groups (with functional cure - 8.3% and without functional cure - 13.2%), making a statistically significant association impossible. This finding does not imply that patients with advanced liver disease cannot achieve functional cure, it only represents that the statistical tests did not have sufficient power to reject the hypothesis that cirrhosis reduces the chances of serological clearance.

It is essential to acknowledge the limitations of this study for a proper interpretation of the results. The retrospective observational design, based on medical record review, carries the risk of incomplete or inconsistent data. The presence of «missing data» in some variables (e.g., 20.7% for the clinical phase) is a limitation that may have impacted the robustness of the analyses. Furthermore, it was noteworthy that 111 patients (18.5% of the initial sample of 600 patients) developed undetectable HBV-DNA during follow-up but without a record of HBsAg testing, highlighting the importance of periodic determination of this biomarker. It is likely that the functional cure rate was higher in the studied population; however, due to the lack of serological confirmation, these patients had to be excluded from the study.

The main limitation lies in the small number of HBsAg clearance events (n=12). This prevented a multivariate analysis using logistic regression, which would have allowed the identification of independent predictors and the evaluation of the interaction between variables. With such a small number of events, the ability to detect statistically significant associations for less prevalent factors or those with more subtle effects is considerably reduced.

Additionally, being a single-center study, the results may not be fully generalizable to other populations of patients with chronic hepatitis B, who may present different genotypic profiles, access to treatments or distinct demographic and clinical characteristics. The follow-up time may be insufficient to capture all clearance events, given the slow and gradual nature of this process in the natural history of the disease.

Despite its limitations, this was the first epidemiological study in Brazil that sought to identify factors associated with the functional cure rate in adult patients with chronic hepatitis B. The findings of this cohort reinforce the importance of regularly monitoring HBV-DNA levels and patient age as potential indicators of a more favorable prognosis regarding HBsAg clearance. Undoubtedly, prospective studies in large specialized centers are needed to better understand the factors associated with the functional cure of chronic hepatitis B.

## CONCLUSION

This study demonstrated an annual HBsAg serological clearance rate of 0.78% and identified older age and low HBV viral load levels as factors associated with functional cure. These results enrich the knowledge about the natural history of chronic hepatitis B and reaffirm the importance of viral suppression for disease control.

## Data Availability

data available upon request

## References

[1] WHO. World Health Organization (2024). Guidelines for the prevention, diagnosis, care and treatment for people with chronic hepatitis B infection.

[2] BRASIL. Ministério da Saúde. Secretaria de Ciência, Tecnologia, Inovação e Complexo da Saúde. Secretaria de Vigilância em Saúde e Ambiente (2023). Protocolo Clínico e Diretrizes Terapêuticas de Hepatite B e Coinfecções.

[3] Lampe E, Fca Mello, Mp Do Espírito-Santo, Cmc Oliveira, Da Bertolini, Nsl Gonçales (2017). Nationwide overview of the distribution of hepatitis B virus genotypes in Brazil: a 1000-sample multicentre study. J Gen Virol.

[4] Xm Song, Ql Li, Guo F, Peng H, Jj Guo (2018). The Effect of ICOS Polymorphism Interactions with HBV Mutations on HBV Subtype Infection Outcomes. Annals of Hepatology.

[5] Shen C, Jiang X, Li M, Luo Y (2023). Hepatitis Virus and Hepatocellular Carcinoma: Recent Advances. Cancers.

[6] Sk Sarin, Kumar M, Gk Lau, Abbas Z, Hl Chan, Cj Chen (2016). Asian-Pacific clinical practice guidelines on the management of hepatitis B: a 2015 update. Hepatol Int.

[7] Fattovich G, Bortolotti F, Donato F (2008). Natural history of chronic hepatitis B: Special emphasis on disease progression and prognostic factors. Journal of Hepatology.

[8] Bj Mcmahon (2009). The Natural History of Chronic Hepatitis B Virus Infection. Hepatology.

[9] As Lok, Zoulim F, Dusheiko G, Mg Ghany (2017). Hepatitis B cure: from discovery to regulatory approval. Hepatology.

[10] Likhitsup A, As Lok (2019). Understanding the Natural History of Hepatitis B Virus Infection and the New Definitions of Cure and the Endpoints of Clinical Trials. Clin Liver Dis.

[11] Wu S, Yi W, Gao Y (2022). Immune Mechanisms Underlying Hepatitis B Surface Antigen Seroclearance in Chronic Hepatitis B Patients With Viral Coinfection. Frontiers in Immunology.

[12] Yh Yeo, Hj Ho, Hi Yang (2019). Associated With Rates of HBsAg Seroclearance in Adults With Chronic HBV Infection: A Systematic Review and Meta- Analysis. Gastroenterology.

[13] Wj Jeng, Yc Chen, Rn Chien, Is Sheen, Yf Liaw (2018). Incidence and predictors of hepatitis B surface antigen seroclearance after cessation of nucleos(t)ide analogue therapy in hepatitis B e antigen-negative chronic hepatitis B. Hepatology.

[14] Zhou K, Contag C, Whitaker E, Terrault N (2019). Spontaneous loss of surface antigen among adults living with chronic hepatitis B virus infection: a systematic review and pooled meta-analyses. Lancet Gastroenterol Hepatol.

[15] Tc tseng, Chiang C, Cj Liu (2023). Low Hepatitis B Core-Related Antigen Levels Correlate Higher Spontaneous Seroclearance of Hepatitis B Surface Antigen in Chronic Hepatitis B Patients With High Hepatitis B Surface Antigen Levels. Gastroenterology.

[16] Marcellin P, Buti M, Krastev Z, Ra De Man, Zeuzem S, Lou L (2014). Kinetics of hepatitis B surface antigen loss in patients with HBeAg-positive chronic hepatitis B treated with tenofovirdisoproxil fumarate. J Hepatol.

[17] Marcellin P, Sh Ahn, Ma X, Fa Caruntu, Wy Tak, Elkashab M (2016). Combination of Tenofovir Disoproxil Fumarate and Peginterferonα-2aIncreases Loss of Hepatitis B Surface Antigen in Patients With Chronic Hepatitis B. Gastroenterology.

[18] WHO (2025). Obesity and overweight.

[19] Manno M, Cammà C, Schepis F, Bassi F, Gelmini R, Giannini F (2004). Natural History of Chronic HBV Carriers in Northern Italy: Morbidity and Mortality After 30 Years. Gastroenterology.

[20] Ys Hsu, Rn Chien, Ct Yeh, Is Sheen, Hy Chiou, Cm Chu (2002). Long-Term Outcome After Spontaneous HBeAg Seroconversion in Patients with Chronic Hepatitis B. Hepatology.

[21] Liu M, Tseng T-C, Dw Jun, Yeh M-L, Trinh H, Glh Wong (2021). Transition rates to cirrhosis and liver cancer by age, gender, disease and treatment status in Asian chronic hepatitis B patients. Hepatol Int.

[22] Na Terrault, Nh Bzowej, Km Chang, Jp Hwang, Mm Jonas, Mh Murad, American Association for the Study of Liver Diseases (2016). AASLD guidelines for treatment of chronic hepatitis B. Hepatology.

[23] Ly Mak, Rwh Hui, Fung J, Liu F, Dkh Wong (2020). Diverse effects of hepatic steatosis on fibrosis progression and functional cure in virologically quiescent chronic hepatitis B. Journal of Hepatology.

[24] Hirode G Choi Hsj, Chen Ch, Su Th Seto Wk (2022). Off-Therapy Response After Nucleos(t)ide Analogue Withdrawal in Patients With Chronic Hepatitis B: An International, Multicenter, Multiethnic Cohort (RETRACT-B Study). Gastroenterology.

[25] Mj Sonneveld, Sm Chiu, Jy Park, Sm Brakenhoff, Kaewdech A (2024). HBV DNA and HBsAg Levels at 24 Weeks Off-Treatment Predict Clinical Relapse and HBsAg Loss in HBeAg-Negative Patients Who Discontinued Antiviral Therapy. Gastroenterology.

[26] Ej Dongelmans, Hirode G, Be Hansen, Wj Jeng, Hla Janssen (2025). Predictors of hepatic flares after nucleos(t)ide analogue cessation - Results of a global cohort study (RETRACT-B study). Journal of Hepatology.

